# Sir Thomas Grainger Stewart

**Published:** 1900-04

**Authors:** 


Sir Thomas Grainger Stewart, M. D., F.R.C.P.E., F. R. S. E., LL.D., died February 3, 1900, at his house, 19 Charlotte Square, Edinburgh, aged 62 years. He was Physician-in-ordinary to the Queen for Scotland, professor of the practice of physic, University of Edinburgh, and president of the British Medical Association at the Edinburgh meeting of 1898. Sir Thomas was distinguished for his researches in diseases of the kidneys, and was an acknowledged authority on this subject. He belonged to an interesting group of medical men fast disappearing and which lent lustre to the guild.

